# WHAT MATTERS FOR COMPLETION OF ADVANCE DIRECTIVES AMONG AMERICAN OLDER ADULTS?

**DOI:** 10.1093/geroni/igz038.2543

**Published:** 2019-11-08

**Authors:** Yifan Lou, Jinyu Liu

**Affiliations:** 1 Columbia University, New York, New York, United States; 2 Columbia University, New York City, New York, United States

## Abstract

Background: Most previous studies consider advance directives as one single outcome, which conceals possible variations of individuals’ decisions on two different advance directives documents–living will (LW) and durable power of attorney for healthcare (PA). To advance the knowledge on advance planning among older adults in the US, this study examined how health status and education are associated with completions of LW and PA and whether such associations vary by age and race. Methods: Data are from the 2016 wave of Health and Retirement Study. Health status was indicated by chronic condition and ADL and IADL functional limitations. Logistic regression model was used to examine how the completions of LW and PA are associated with health and education variables respectively. Interaction terms were created to test the moderating effects of race and age. Results: The analysis results show that older adults with higher IADL functional limitation and more education were more likely to complete completing PA or LW. Being white and higher age will increase the probability for an older adult to complete PA, whereas the older adults with heart problem were more likely to complete LW. The association between IADL/ADL and PA was stronger in white than other racial groups, and the association between IADL and LW was stronger in young-old than the oldest-old. Conclusion: The findings highlight the importance of examining the completions of two advance directive documents respectively and indicate the necessity of developing distinct and concrete strategies to promote the completions of PA and LW.

